# Sexual Dysfunction, Depression and Quality of Life in Patients With HIV Infection

**Published:** 2013

**Authors:** Mahmood Amini Lari, Hosain Faramarzi, Mesbah Shams, Maryam Marzban, Hasan Joulaei

**Affiliations:** 1 Shiraz HIV/AIDS Research Center, Shiraz University of Medical Sciences, Shiraz, Iran.; 2 Namazi Hospital , endocrine & Metabolism research center, Shiraz University of Medical Sciences, Shiraz-Iran.; 3 School of public health, Bushehr University of Medical Sciences Bushehr , Iran.; 4 Health policy research center Shiraz University of Medical Sciences, Shiraz, Iran.

**Keywords:** Depression, HIV, Quality of life, SF36, Sexual function

## Abstract

**Objective**: In Iran, psychological aspect of HIV infection is poorly understood. The purposes of this study were to evaluate sexual dysfunction, depression rate and health-related quality of life and evaluate the association between sexual dysfunction, depression and quality of life in a group of HIV^+^ subjects in Shiraz, Iran.

**Methods**: In this cross-sectional study, 278 male HIV-positive patients who had referred to voluntary counseling and testing and methadone maintenance therapy centers were recruited based on convenience sampling from May to October 2010. The purpose of the study was explained and interested individuals provided informed consent and completed validated questionnaires [Medical Outcomes Study Short Form-36 (SF-36(, Brief Male Sexual Function Inventory (BMSFI), Beck Depression Inventory-short form(BDI)] to assess overall health related quality of life (HRQOL), sexual function, and depression.

**Results**: Average age of the participants was 34.9 ± 10.7 years and 37.5% were severely depressed. Ordinal logistic regression indicated that sexual drive (-0.15; CI: -0.28 to -0.027), ejaculation (-1.91, CI: -2.71 to -1.12), and problem assessment (-0.098, CI: -0.17 to -0.02) had significant effect on depression type. Depression was significantly correlated with poorer quality of life in all domains. Pearson’s correlation coefficients between the BMSFI and the domains of SF-36 indicated that sexual drive (r= 0.215), ejaculation (r= 0.297) and problem assessment (r= 0.213) were significantly correlated with emotional wellbeing.

**Conclusion**: Sexual function and depression showed association with quality of life. Effective treatment of depression and sexual function may improve the quality of life of HIV-infected person.

**Declaration of interest**: None.

## Introduction

Sexual dysfunction has been widely reported among men living with HIV infection and also HIV uninfected men. Studies demonstrated that between 13% and 74% of HIV+ men in the highly active antiretroviral therapy suffering from sexual dysfunction (1). In Iran, although, persons with HIV infection usually suffer from sexual dysfunction, but its frequency and manifestations in this population is not well known (2). Both organic and psychological factors have been identified as the causal factors for sexual dysfunction (3). Indeed, poor emotional and psychological functions among people living with HIV, may lead to sexual dysfunction (4). The psychological aspects of sexual dysfunction may take on an added dimension in patients with HIV infection. Regardless the mode of transmission, HIV-positive patients may be upset with the risk of transmitting HIV to others. So, psychological issues as a cause or an effect of sexual dysfunction should be considered by clinicians for better handling of HIV+ patients (3). Sexual dysfunctions moreover are conceptualized as one component of sexual health, which is an essential element of overall health related quality of life (HRQOL). Sexual health encompasses the possibility of having pleasurable and safe sexual experiences (5).

Sexual dysfunction has been widely reported among men living with HIV infection and also HIV uninfected men. Studies demonstrated that between 13% and 74% of HIV+ men in the highly active antiretroviral therapy suffering from sexual dysfunction (1). In Iran, although, persons with HIV infection usually suffer from sexual dysfunction, but its frequency and manifestations in this population is not well known (2). Both organic and psychological factors have been identified as the causal factors for sexual dysfunction (3). Indeed, poor emotional and psychological functions among people living with HIV, may lead to sexual dysfunction (4).

The psychological aspects of sexual dysfunction may take on an added dimension in patients with HIV infection. Regardless the mode of transmission, HIV-positive patients may be upset with the risk of transmitting HIV to others. So, psychological issues as a cause or an effect of sexual dysfunction should be considered by clinicians for better handling of HIV+ patients (3). Sexual dysfunctions moreover are conceptualized as one component of sexual health, which is an essential element of overall health related quality of life (HRQOL). Sexual health encompasses the possibility of having pleasurable and safe sexual experiences (5).

*Depression*

Not only physical manifestations, but psychological health is also negatively affected in individuals living with HIV/AIDS. The prevalence of depression in the HIV population remains high and should be continually addressed. Among individuals with HIV disease, major depression is a frequently observed psychiatric disorder. However, the relationships between HIV and depression are very complex and difficult to assess (6).

 The symptoms of depression are similar in HIV-infected and non-infected patients, but patients with HIV infection may more frequently have sleep and appetite disturbances. Patients with a history of depression, homosexual men, women, and intravenous drug abusers are among HIV-infected individuals who may be at increased risk for depression (7). As the number of persons living with HIV continues to grow, it is essential to explore the prevalence of HIV-related depression, as well as the factors that contribute to the depression. Some studies indicate a large range of prevalence rates of depression in HIV, with reports ranging from 5% to 45% (6-10). 

*Quality of life*

Quality of life is an important component in the evaluation of the well-being of HIV-infected patients (11). Although medical aspects of HIV/AIDS are well documented, self-reported HRQOL measures are less well understood, particularly among those patients with advanced disease (12). HRQOL refers to how well a person functions and to his or her perceptions of well-being in the physical, mental, and social domains of life (13). Assessing HRQOL is useful for documenting the burden of chronic disease, following changes in health over time, and comparing the overall effects of treatments (14). Quality of life is an important component in the evaluation of patients well-being following HIV infection. Improving quality of life is a major goal in treating individuals infected with HIV. Measuring HRQOL is accepted as capturing the overall impacts of interventions on patients’ functioning and wellbeing, which goes beyond the interpretability and clinical meaning of immunologic and virologic markers (15). Many studies have investigated HRQOL in patients with HIV (16-18), and some have assayed the association between HRQOL and depression (19). As Felton et al. have shown HRQOL is the single most important predictor of depression. Another study demonstrated that reduction in depression lead to a significant improvement in HRQOL with the exception of work and financial functioning (20).

To the best of our knowledge, no studies have reported depression rate, sexual dysfunction, and HRQOL of people living with HIV and their associations to each other in Iran. This paper describes sexual dysfunction, depression and HRQOL and evaluates the impact of sexual dysfunction on depression and quality of life in a group of HIV-positive subjects in Shiraz, Iran. 

## Materials and Methods

*Sample*

From 315 participants a total of 278 HIV-positive patients were recruited based on convenience sampling from May to October 2010 with a response rate of over 88 percent. Data were collected from Voluntary Counseling and Testing Center (VCT) and MMT center, related to Shiraz University of Medical Sciences, Shiraz, Iran. 

The purposes of the study were explained for all participants and interested individuals provided informed consent and completed a questionnaire detailing socio-demographic and HIV related variables and validated questionnaires to assess sexual function, depression and health related quality of life. This procedure was administered to the subjects when they came to the VCT and MMT centers, Shiraz, Iran.

*Tools: SF-36*

To assess HRQOL the Medical Outcomes Study Short Form-36 (SF-36) was used. This self-administered includes one multi-item scale that assesses eight health concepts: limitations in physical activities because of health problems (e.g., walking, dressing), limitations in social activities because of physical or emotional problems (e.g., meeting friends), limitations in usual role activities because of physical health problems, bodily pain (presence of pain and limitations due to pain), general mental health (psychological distress and well-being), limitations in usual role activities because of emotional problems, vitality (loss of energy or presence of fatigue), and general health perceptions (5). Each HRQOL domain is given a score ranging from 0 to 100, with higher scores indicative of better quality of life (21). In this study quality of life data were collected through computerized scoring software.

*Brief Male Sexual Function Inventory (BMSFI)*

To assess sexual dysfunction, the Brief Male Sexual Function Inventor (BMFSI) was used. This instrument consists of 11 questions with 5 possible responses scored on a scale of 0 (worst) to 4 (best). These questions are summarized into 5 domains, including sexual drive (questions 1 and 2), erectile function (questions 3 to 5), ejaculatory function (questions 6 and 7), problem assessment (questions 8 to 10) and overall sexual satisfaction (question 11). The value of each domain reflects the simple algebraic sum of the scores of the underlying question(s) with lower domain scores indicate more impaired sexual function (22).

*Beck Depression Inventory-short form (BDI)*

The Beck Depression Inventory II (BDI-II) was used to assess the prevalence and severity of depressive symptoms. The BDI-II has shown high validity and reliability in measuring depressive symptoms. Respondents were required to rate 21 items from 0 to 3 according to how they had felt during the previous 2 weeks. The BDI-II focuses on both the cognitive-affective symptoms of depression, such as pessimism and diminished self-esteem, and the somatic symptoms of depression such as weight loss (23). The questionnaires described above all have been validated in previous studies (23, 24).

*Data Analysis*

The data were analyzed using the Statistical Package for the Social Sciences (SPSS) for Windows, Version 18.0. We carried out a simple descriptive analysis of the patients’ perception of their quality of life, sexual dysfunction and depression and demographic variables. The Pearson's correlation coefficients (r) and ordinal logistic regression were used for analysis.

## Results

*Study Participation and Participant Characteristics*

A total of 278 patients were included in the study. All patients included in this study had received a diagnosis of HIV infection. Patient characteristics are presented in table 1. The mean age was 37.4 (SD±7.4) years, all of them were male and 84.2% were unemployed. 

**Table 1:** Characteristic of 278 HIV-seropositive patients in Shiraz, Iran

**Characteristic**	**Frequency**	**Percentage**
**Age group (in year)**		
25-30 30-40 41 and older Missing Data	025 132 086 035	09% 47.5% 30.9% 12%
**Marital statues**		
Single Married Separated Divorced Live with permanent partner Live with temporary partner Missing Data	119 094 022 009 005 002 027	42.8% 33.8% 07.9% 03.2% 01.8% 00.7% 09.7%
**Employment status**		
Un Employed Employed	234 044	84.2% 15.8%
**Level of education**		
Illiterate Primary school and secondary school High school Higher than high school diploma Missing Data	030 123 057 024 044	10.8% 44.2% 20.5% 08.7% 15%
**Cigarette smoking**		
Yes No Missing Data	227 043 008	81.7% 15.5% 02%
**Methadone maintenance therapy**		
Yes No Missing Data	114 162 002	41% 58.3% 00.7%
**Entering the AIDS stage**		
Yes No Missing Data	188 084 006	67.6% 30.2% 00.2%

Mean, Standard deviation and score range for five domains of BMSFI are presented at table 2.

**Figure 1:**
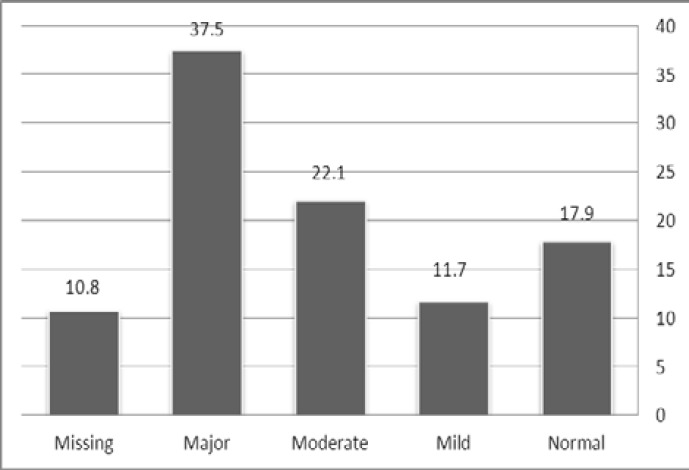
Severity of depression among 278 HIV-infected patients studied

Among the 278 HIV-seropositive subjects enrolled, 211 (75.9%) patients were diagnosed to be depressed and 79(37.5%) were severely depressed. The prevalence of diagnosed depression among the population of 278 HIV-positive patients appears in figure 1.

*Pattern of drug abuse and depression*

One-hundred sixteen (42%) were receiving methadone at the time of study. Among non methadone users (162 cases) the illicit substances abused were heroin (49 cases, 30.2%), opium (19 cases, 11.5%), marijuana (3 cases, 2.1%), and a combination of multiple illicit drugs (41 cases, 25%). Substances of abuse were not defined in52 cases (31.2%). According to the univariate analysis, there was no significant association between depression and pattern of drug substance abuse (p=0.103). There was no statistically significant difference regarding depression rate between those who were receiving methadone and others (p= 0.142).

**Table 2:** Mean, standard deviation and score range for five domains of BMSFI among 237 HIV-seropositive patients

	**Range**	**Mean (±SD)**
**Sexual drive**	0-08	3.7 (±1.92)
**Ejaculation**	0-12	6.36 (±2.7)
**Erection**	0 - 8	5.2 (±2.19)
**Problem assessment**	0-12	8.04 (±3.63)
**Overall satisfaction**	0-04	2.42 (±1.05)

*Effect of sexual dysfunction on depression*

Firstly, according to depression scores, we categorized the subjects into two groups. A BDI-II score of more than 18 was chosen as the cut-off point to distinguish patients with moderate to severe depression from those with normal or only minimally elevated depression scores.

 Then, the association between five domains of the BMSFI and depression was investigated using binary logistic regression. The results indicated that sexual drive (OR= 0.84; CI: 0.71- 0.99) and problem assessment (OR=0.883; CI: 0.80-0.97) had significant effect on the presence of depression (Table 3).

**Table 3:** Effect of sexual dysfunction on depression among 273 HIV^+^ men

**Sexual function**	**Odds Ratio**	**Confidence Interval**	**P-Value**
**Sexual drive**	0.84	0.71 -0.99	0.038
**Erection**	0.91	0.80-1.03	0.142
**Ejaculation**	0.86	0.73-1.02	0.092
**Problem assessment**	0.883	0.80-0.97	0.011
**Overall satisfaction**	0.98	0.95-1.012	0.25

*According to the results of evaluating sexual dysfunction, the mean and standard deviation of 8 health concepts of SF36 were as follow:* limitations in physical activities because of health problems (e.g., walking, dressing) (32.6±25), limitations in social activities because of physical or emotional problems (e.g., meeting friends) (45.6±43.7), limitations in usual role activities because of physical health problems (55.11±55), bodily pain (presence of pain and limitations due to pain) (51,31±50), general mental health (psychological distress and well-being) (46.3±43.7), limitations in usual role activities because of emotional problems (42.5± 40.7), vitality (loss of energy or presence of fatigue)(43.08±45), and general health perceptions (48.3± 50).

*Correlation of BMSFI with SF-36 scales*

The Pearson’s r correlation coefficients between the BMSFI and the domains of SF-36 in all 278 patients indicated that emotional well being was correlated with sexual drive (r= 0.215, p= 0.006), ejaculation (r= 0.297, p< 0.0001) and problem assessment (r= 0.213, p= 0.004). Likewise, significant associations were detected between general health and ejaculation (r= 0.155, p= 0.046) and between social functioning and problem assessment (r= 0.192, p= 0.01).

*Correlation of depression with SF-36 scales*

Table 4 shows the Pearson’s r correlation coefficients between depression and the SF-36 composite subscales. There was statistical significance between all domains of the SF-36 and type of depressive disorder and emotional well being had the strongest correlation was observed with depression type (r= 0.452). 

**Table 4:** The Pearson’s r correlation coefficients between the type of depression and SF36 subscales among 278 HIV^+^ patients

	**r**	**p**
**Physical functioning**	0.386	< 0.0001
**Role limitation due to physical health**	0.285	< 0.0001
**Pain**	0.443	< 0.0001
**General health**	0.220	0.001
**Energy/fatigue**	0.413	< 0.0001
**Social functioning**	0.408	< 0.0001
**Role** **limitation due to emotional problems**	0.324	< 0.0001
**Emotional well being**	0.452	< 0.0001

## Discussion

In Iran the epidemiology of sexual dysfunction in HIV/ADS patients and its relationship to quality of life and depression in this population are poorly understood. The purpose of our study was to determine the prevalence of depression and sexual dysfunction and to measure HRQOL and to evaluate the impact of sexual dysfunction on depression and quality of life in a group of HIV-positive subjects in Shiraz, Iran. 

Sexual health is defined in terms of well-being, but is challenged by the social, cultural and economic realities faced by men with HIV (25). Sexual dysfunctions are considered as main part of sexual health, which is an essential element of overall HRQOL (5). Depressive disorders are common among 20% to 32% of people with HIV but often are not identified well (26). In our study most of the patients were depressed. In similar studies held in Denmark and Australia, symptoms of depression (i.e., BDI> 14) have been observed among most HIV-infected patients (23-27). The results of present study revealed that the rate of depression was higher in comparison to the mentioned studies. The findings approve that depression is less diagnosed and treated in HIV-infected patients. Sexual dysfunction is often implicated in depression and anxiety disorders and it is more common in depressed patients (28). Most patients suffering from mild, moderate, or major forms of depression experience sexual dysfunction simultaneously (29). 

For a long time, the sexual behavior of HIV-infected persons did not receive any serious attention (30). Our result showed that depressed patients significantly had lower scores in some domains of BMFSI and by increasing score of sexual drive and problem assessment the depression status become well. 

Depression adversely affects QOL, and that effective treatment of depression may dramatically improve the QOL of HIV-infected persons (31), and it is related to most indices of quality of life among HIV-positive adults. Thus, in HIV-infected persons, a subject’s experience of depressive symptoms is a good predictor of poor QOL. Depression adversely affects QOL, and that effective treatment of depression may dramatically improve the QOL of HIV-infected person (31).

Our study indicated that depression was significantly correlated with all domains of quality of life. In a Nigerian study, patients with a clinical diagnosis of depression had significantly lower HRQOL scores in all domains except ‘social relationships (10). Some other studies demonstrated association between depression and quality of life among HIV+ patients (19, 20).

On the other hand sexual function has been recognized as a crucial component of health, in fact experience of sexual dysfunction is generally associated with poor quality of life (32). According to our findings about the relationships between some components of QOL and some domains of BMSFI, by improving sexual function HRQL can increased and this should be an important goal for clinicians involved in the care of HIV-infected individuals.

The present study is limited by its lack of conscious participants because of their poor socio- economical status such as low educational level, unemployment, poor social and family support and addiction and also the content of BMSFI, so data collection was so hard in this group and missing data is expected. These variables have not been taken into account though available literatures suggest that they influence quality of life significantly, sexual dysfunction and depression. Age, marital status and education that are significantly associated with quality of life, have also not been controlled in the present study. Due to the cross-sectional design, the findings point more toward an association rather than cause and effect. Various psychological symptoms are associated with HIV infection and associated with quality of life, sexual dysfunction and depression; these factors are not taking into account in the present study too.

In conclusion we suggest future studies to evaluate our variables in female HIV-infected patients and comparison according to gender differences, also we offer administration randomized clinical trial to evaluate effect of endocrine treatment like testosterone therapy on sexual dysfunction for promoting health related quality of life in people who live with HIV. 

## Authors’ Contributions

MAL designed the evaluation and drafted the manuscript. HF participated in clinical data collection. MSh performed the statistical analysis. MM re-evaluated the clinical data and HJ revised the manuscript. All authors read and approved the final manuscript.
